# Object Detection on Road: Vehicle’s Detection Based on Re-Training Models on NVIDIA-Jetson Platform

**DOI:** 10.3390/jimaging12010020

**Published:** 2026-01-01

**Authors:** Sleiter Ramos-Sanchez, Jinmi Lezama, Ricardo Yauri, Joyce Zevallos

**Affiliations:** Grupo de Investigación en Circuitos y Sistemas Electrónicos de Alta Frecuencia, Universidad Nacional Tecnológica de Lima Sur, Villa El Salvador, Lima 15834, Peru; 2419010015@untels.edu.pe (S.R.-S.); ryauri@untels.edu.pe (R.Y.); 2211110288@untels.edu.pe (J.Z.)

**Keywords:** object detection, embedded platform, computer vision, NVIDIA Jetson

## Abstract

The increasing use of artificial intelligence (AI) and deep learning (DL) techniques has driven advances in vehicle classification and detection applications for embedded devices with deployment constraints due to computational cost and response time. In the case of urban environments with high traffic congestion, such as the city of Lima, it is important to determine the trade-off between model accuracy, type of embedded system, and the dataset used. This study was developed using a methodology adapted from the CRISP-DM approach, which included the acquisition of traffic videos in the city of Lima, their segmentation, and manual labeling. Subsequently, three SSD-based detection models (MobileNetV1-SSD, MobileNetV2-SSD-Lite, and VGG16-SSD) were trained on the NVIDIA Jetson Orin NX 16 GB platform. The results show that the VGG16-SSD model achieved the highest average precision (mAP ≈90.7%), with a longer training time, while the MobileNetV1-SSD (512×512) model achieved comparable performance (mAP ≈90.4%) with a shorter time. Additionally, data augmentation through contrast adjustment improved the detection of minority classes such as Tuk-tuk and Motorcycle. The results indicate that, among the evaluated models, MobileNetV1-SSD (512×512) achieved the best balance between accuracy and computational load for its implementation in ADAS embedded systems in congested urban environments.

## 1. Introduction

Currently, the use of artificial intelligence (AI) techniques has transformed perception systems for vehicles, driving advances in object detection and driving assistance worldwide [1,2]. Training deep learning (DL) models requires significant computational and communication resources, making it necessary to analyze the trade-off between computational cost and accuracy for deployment in environments with hardware limitations [3,4]. Furthermore, the integration of these techniques with smart vehicles can generate massive volumes of data, increasing transmission and storage costs. In addition, the growth of the automotive market and public policies aimed at safety have increased interest in advanced driver assistance systems (ADAS), but the heterogeneity of infrastructure (insufficient signage, non-homogeneous roads) and the diversity of vehicles present particular challenges for detectors trained with country-specific datasets [5,6]. The city of Lima, which has highly congested traffic conditions and obstacles on the roads, provides a realistic and challenging test bench for the evaluation of embedded computer vision models, considering that according to the TomTom platform report [7], Lima ranks seventh worldwide in terms of traffic congestion, with a level of 47%, and a loss of 150 h per year during peak hours.

The research described in this article analyzes the performance of detection architectures, evaluating the best compromise between accuracy and efficiency in an embedded device such as the Jetson Nano-Orin in the context of Peruvian traffic. The problem identified focuses on evaluating and quantifying which vehicle detection models can be executed in real time and with limited resources on an embedded device (NVIDIA Jetson Orin NX 16 GB) without sacrificing the minimum accuracy required for driver assistance applications. This involves evaluating not only detection accuracy in real-world scenarios in Lima, but also the requirements and methodologies for deploying detection techniques.

Previous work has shown that lightweight architectures such as MobileNet-SSD and SSD-Lite variants can be compared in terms of metrics such as accuracy and efficiency [8,9] in similar application contexts, while heavier backbones (such as VGG16) tend to provide higher mean average precision (mAP) at the cost of higher latency and consumption [10,11]. In addition, recent studies on deployment in embedded systems highlight the importance of optimizations (quantization, pruning, acceleration by TensorRT) to achieve realistic performance on Jetson platforms [12,13]. However, most evaluations are performed with datasets and conditions that differ from the Peruvian context of this research. Therefore, an empirical and comparative evaluation under real conditions and on specific hardware is required.

The methodology follows an adapted CRISP-DM/KDD approach, which includes the collection and annotation of a representative dataset of Lima traffic, preprocessing, and comparative field evaluation using metrics based on the confusion matrix [14,15]. The objective of the research is to evaluate and compare the performance of MobileNetV1-SSD, MobileNetV2-SSD-Lite, and VGG16-SSD detection models on NVIDIA Jetson Orin NX 16 GB for the detection of vehicles characteristic of Peru in real traffic conditions in Lima. To this end, a series of steps are carried out, including the collection and annotation of a set of images and videos representative of the vehicle fleet, the implementation of the three target architectures on the Jetson Orin, the deployment of models, and the comparison and verification of results with experiments based on new datasets. Therefore, an evaluation methodology is described that allows researchers to reproduce the mechanisms for choosing the most appropriate architecture and configuration for ADAS with light autonomous vehicles in contexts similar to Peru. Based on the above, the contributions of the research are as follows:

- Generation of a representative annotated dataset that can be made available to the community for replicability;
- Reproducible evaluation of embedded detection models for Lima’s vehicle fleet and urban conditions;
- Training and implementation of models on a Jetson Orin NX 16 GB embedded platform;
- Provides recommendations based on analysis of model accuracy and preprocessing strategies for deployment on embedded devices.

This article is organized as follows. Section 1 provides an introduction based on the context, description of the problem, contribution, and motivation. Section 2 describes related work and the state of the art in embedded detection and deployment architectures. Section 3 details the methodology for data collection, annotation, implementation procedure, and metrics used. Section 4 presents the results and discussion. Finally, Section 5 offers conclusions, limitations of the study, and future work.

## 2. Related Works

The autonomous navigation of vehicles in streets and avenues has become an important area of research in computer vision, robotics, and intelligent systems [16]. Recent advances have integrated multiple autonomous driving techniques, ranging from trajectory planning [17] and control systems [18] to the intensive use of perception algorithms based on computer vision, which are necessary to interpret complex urban environments [19,20]. In this context, the key focus of this research is determining the efficiency of these systems for vehicle localization, tracking, and detection in complex scenarios through the combination of optimized architectures for developing autonomous navigation systems and their application in urban mobility.

In the field of computer vision, object detection applied to vehicles has evolved from classical methods based on machine learning and distance or radiofrequency sensors to modern high-performance architectures. In this domain, models such as SSD and EfficientDet have been evaluated and compared considering metrics such as precision, processing time, and detection speed—critical aspects in autonomous driving scenarios and advanced driver assistance systems (ADAS), where proactive responses are necessary to prevent vehicular accidents [21,22]. On the other hand, other R-CNN-based approaches have stood out for their accuracy, while lightweight models such as YOLO have demonstrated their applicability in real-time environments for terrestrial vehicle detection [23,24]. These techniques are evaluated using benchmark datasets such as KITTI, COCO, and Cityscapes, which have enabled measurement of detector performance against specific traffic elements, such as lighting variability and high object density [25,26].

The integration of lightweight classification and detection models optimized for embedded devices has gained importance in recent years due to the need to implement real-time detection systems on hardware platforms with limited resources. Architectures such as SSD-MobileNet have been studied for their low number of parameters and reduced computational requirements, being integrated into detectors for embedded devices in computer vision scenarios [27,28]. Furthermore, research has demonstrated that such models, when deployed on low-power accelerators like Raspberry Pi, face the challenge of the trade-off between latency and precision [29]. Similarly, their integration into devices such as the Jetson Nano has been used for vehicle detection, evaluating model performance based on inference times and energy consumption [9,30]. In this context, the literature reports different optimization strategies, such as quantization, pruning, and the use of embedded accelerators in critical applications such as ADAS and autonomous systems.

The use of NVIDIA Jetson platforms for real-time vision has been widely utilized in recent years due to their capability to execute detection models in embedded environments with limited resources. Various studies have evaluated the performance of devices such as Jetson Nano and Jetson Orin NX, revealing significant differences in energy consumption, latency, and processing capacity, which guide hardware selection according to model complexity and its application [31,32,33]. To improve efficiency, tools such as TensorRT have been employed for the optimization of deep neural networks through quantization and precision reduction techniques [34,35].

## 3. Materials and Methods

**Figure 1:**
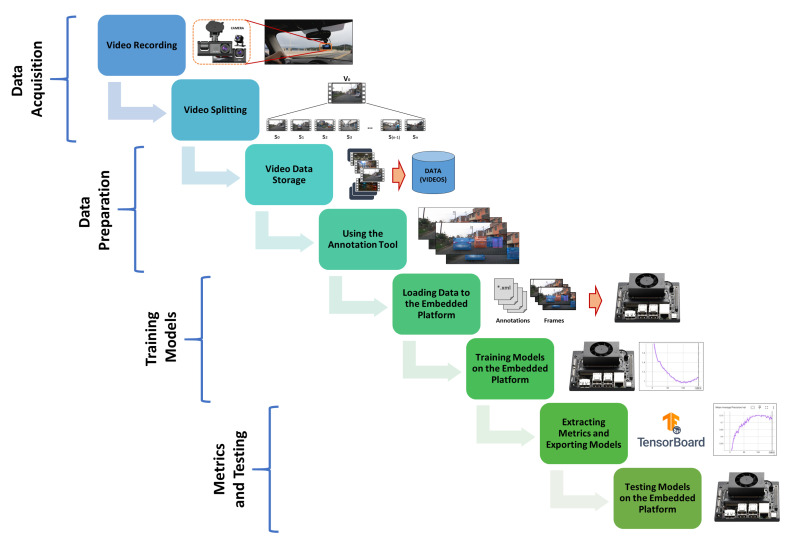
Flowchart of the implementation used on the embedded platform.

The general structure of the work is summarized graphically in Figure 1, from the video acquisition stage of a vehicle in motion in a traffic environment in the city of Lima, through the training of three deep learning models used in computer vision, to the live testing of object detection for moving vehicles. The implementation has been tested and mounted on a vehicle to traverse the streets of Lima, utilizing the NVIDIA embedded platform, Jetson Orin NX with 16 GB, and a webcam as the data input source for the deployed models.

### 3.1. System Architecture

The process shown in Figure 1 consists of the following steps. i. Video Recording: Vehicles are captured in a traffic environment using a dashcam mounted on a vehicle. ii. Video Splitting: Information of interest is extracted and the videos are segmented to facilitate data labeling. iii. Video Data Storage: The selected video segments are aggregated to generate video data ready for annotation. iv. Using the Annotation Tool: Manual labeling is performed using bounding boxes and identifying the classes involved in each frame of the videos. v. Loading Data to the Embedded Platform: The annotations performed and exported in Pascal-VOC format as *.xml files and the extracted video frames in *.png format are loaded onto the Jetson. vi. Training Models on the Embedded Platform: Three models based on SSD as the backbone are re-trained on the embedded platform. vii. Extracting Metrics and Exporting Models: *.tfevents Files are extracted for interpretation using TensorBoard and visualizing metrics such as Average Precision (AP) for each class and mAP, which is the average across classes, in addition to generating *.onnx files for each trained model. viii. Testing Models on the Embedded Platform: DetecNet is used to load the exported models and object detection tests are performed on the Jetson.

### 3.2. Data Acquisition

**Figure 2:**
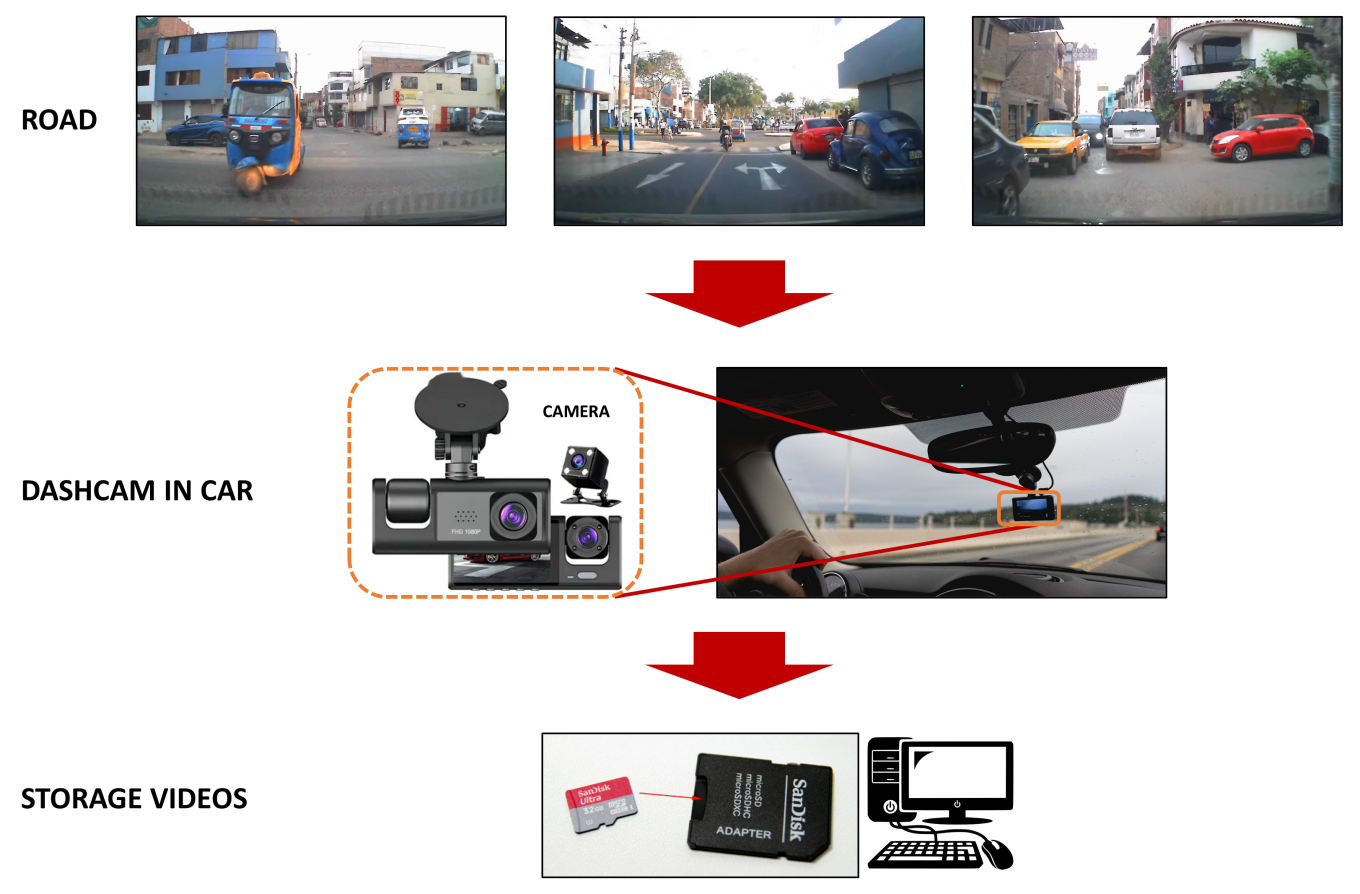
Diagram to record and store videos from the road.

The video data capture scheme was performed using a dashcam installed on an automobile, which captures videos of objects (defined classes) on the road through the car’s windshield, as shown in Figure 2. The videos recorded from the route on the road in an urban environment, specifically in the San Juan de Miraflores district, Lima, Peru, are stored on a micro-SD memory card and later extracted on a desktop computer for the segmentation stage.

**Figure 3:**
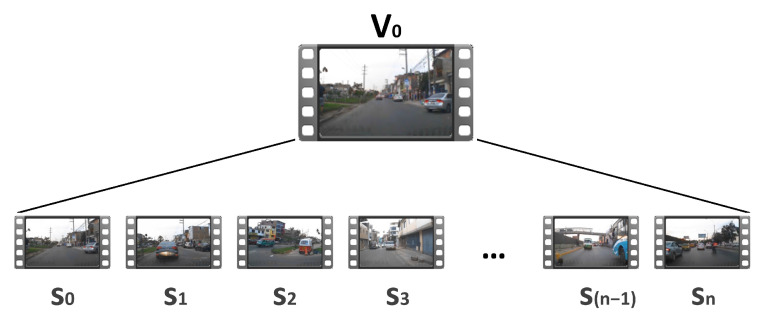
Segmentation of recorded videos into small segments.

The captured videos have been segmented, as shown in Figure 3. The recordings obtained during the urban route consist of videos with a duration of 3 min, comprising a total of n+1 video segments, with each segment having a duration of tf seconds and containing *m* frames per second. For example, in Figure 3, video $V_{0}$ is composed of segments $V_{0}=S_{0}+S_{1}+S_{2}+\ldots +S_{n}$.

**Figure 4:**
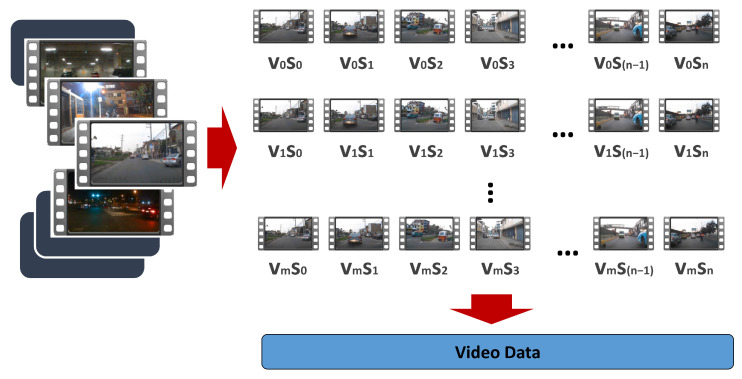
Construction of video data into small segments.

Figure 4 presents a general overview of all video segments collected during the acquisition stage. Only some of the segments have been considered for the dataset; segments have been removed if, for example, they contain very few objects of interest (defined classes). Considering the number of images existing in the selected video dataset, the total number of images for the dataset is obtained by calculating $nf_{g}=tf_{r}\times nf_{r}\times m_{fps}$, where nf is the number of segments, tf=20 s (duration of a video segment), and $m_{fps}=30$ (frames per second). This yields a total of $nf_{g}=3000$ images for the dataset.

### 3.3. Data Preparation

The prepared dataset of 3000 images was annotated using the Computer Vision Annotation Tool (CVAT) [36]. The tool facilitates the creation of labels for each instance of the defined classes using bounding boxes and provides an interpolation mode that alleviates the need to redraw each bounding box for adjacent frames. After completing the annotation of all dataset images, the tool allows exporting in different formats according to the project requirements. For this project, the PASCAL-VOC format [37] was employed, with annotations exported in *.xml format, along with images from video segments that contain the positions of bounding boxes for the previously defined classes of interest.

**Figure 5:**
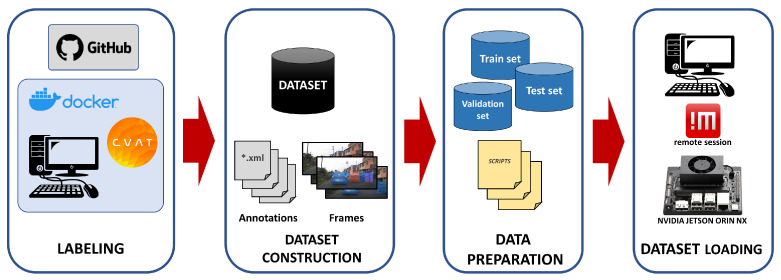
Diagram for loading dataset on the Jetson platform.

A scheme for data preparation and loading onto the embedded platform is shown in Figure 5, which is used after the data acquisition stage mentioned in the previous subsection.

The diagram in Figure 5 consists of four stages: i. Labeling—annotation using the CVAT annotation tool on a computer using Docker; ii. Dataset Construction—exporting the images and annotations for each image; iii. Data Preparation—segmenting the data to obtain three datasets: training, validation, and test; iv. Dataset Loading—importing the data onto the embedded platform using a remote access tool (NoMachine) to the Jetson to facilitate file upload or download.

The distribution of object classes in the 3000 labeled images with seven defined classes (Car, Tuk-tuk, Suv, Motorcycle, Bus, Pedestrian, and Van) is shown in Table 1, with a total of 14,012 objects across all labeled images.

**Figure 6:**
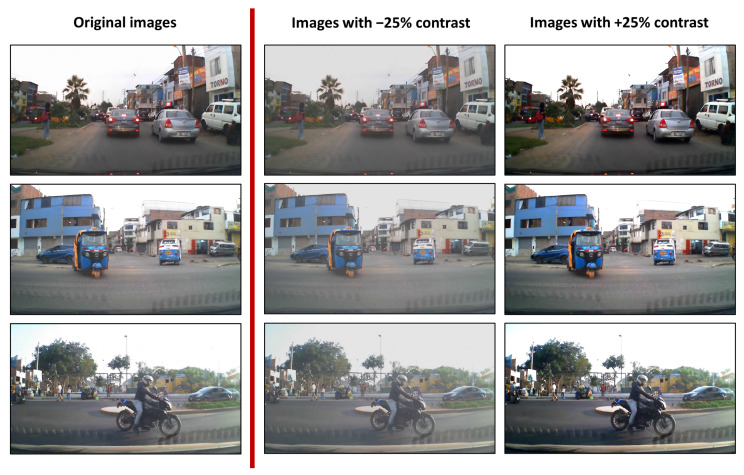
Data augmentation by adjusting the dataset’s contrast.

Data augmentation was performed by adjusting the contrast for each frame of the dataset, as shown in Figure 6.

By executing a script that increases and decreases the contrast by ±25% for each image, the new dataset was tripled with respect to the number of images in the initial dataset. To obtain the new annotations, reference files extracted from the CVAT annotation tool were used, duplicating the annotations from the original image but referencing the new images with adjusted contrast. This was done because the information corresponding to the location of the bounding boxes was not modified.

For both datasets of 3000 and 9000 images (dataset with contrast adjustment), a two-level dataset split was performed to obtain training, test, and validation sets. At the first level, the dataset was divided into 85% and 15%, with the first percentage allocated to a temporary dataset and the latter to the test set. At the second level, the temporary dataset was further split, with 20% for validation and the remainder for the training set. This resulted in a final training set of 68%, a validation set of 17%, and a test set of 15% with respect to the total number of images in the dataset before the split levels.

The distribution of objects across the three subsets for both datasets is shown in Table 2.

The split percentages of the dataset to obtain the three subsets do not necessarily correspond to the multiplicative factor for the distribution of objects from each class shown in Table 2.

### 3.4. Deep Learning Models

**Figure 7:**
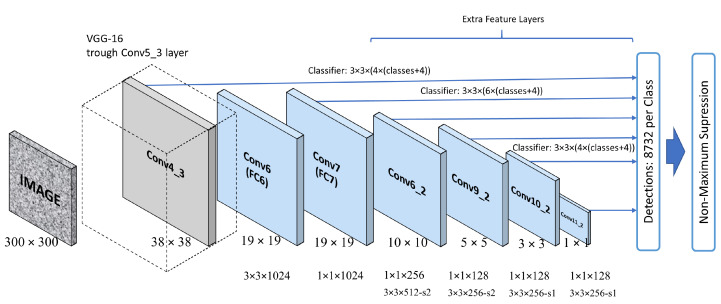
Architecture of the Single-Shot-Detector model [38].

In the present work, three computer vision models have been explored—MobileNetV1-SSD, MobileNetV2-SSD-Lite, and VGG16-SSD—which are based on the Single-Shot-Detector (SSD) network architecture for object detection, proposed by Liu [38], as shown in Figure 7.

SSD is a fast and efficient model for real-time tasks and applications such as object detection in videos. It is capable of detecting objects of different sizes in the same image since it makes predictions at different layers of the network. Additionally, SSD performs post-processing because there can be multiple bounding boxes for the same object. It uses a technique called NMS (Non-Maximum Suppression) to select the best bounding box for each object, eliminating redundant bounding boxes from the same instance.

The three architectures mentioned possess a backbone, which is the part of the architecture that extracts features from images. They are based on MobileNetV1, MobileNetV2, and VGG16, with the first two being lightweight and fast architectures for devices with limited resources, while the latter is more complex and accurate compared to other lightweight models. The three models were retrained on the NVIDIA Jetson Orin NX embedded platform using data from both collected datasets of 3000 and 9000 images.

### 3.5. Training Models

Model training was performed on the same embedded platform, using the Github repository called jetson-inference [39] as a reference and following the documentation that describes the training stage.

In the file structure for the object detection problem based on the SSD model, there is a script for performing training, which receives several arguments that can be modified as needed. For the development of this work, the arguments and values shown in Table 3 were considered.

The pretrained-ssd values are the names of .pth files that were obtained from another repository called pytorch-ssd [40], which were used as the basis for retraining the three models mentioned previously in Section 3.4.

In addition to the eight arguments listed, the argument –validation-mean-ap was employed to compute, at each –validation-epochs, the Average Precision (AP) metric for each class defined in the detection problem, and the mean Average Precision (mAP) as the average of the previous results.

It is worth noting that for the resolution argument, the value of 300×300 was used for all three models, and the value of 512×512 was only used for the MobileNetV1-SSD model reported in the results section.

## 4. Results and Discussion

**Figure 8:**
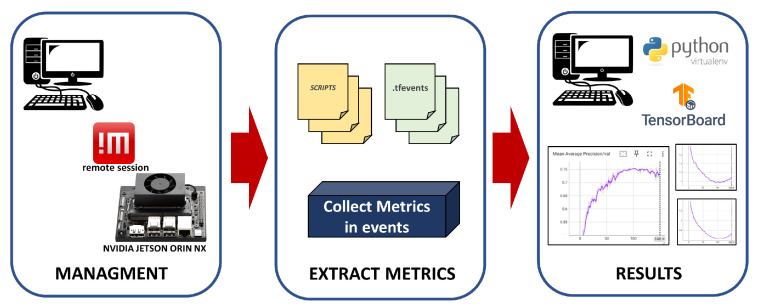
Extract metrics from Jetson platform.

A diagram for the collection and extraction of metrics from the embedded platform to a computer that manages the Jetson is shown in Figure 8.

The diagram consists of three stages. i. Management: Here, the embedded platform is remotely managed from a computer using the NoMachine application. ii. Extract metrics: During training, with the arguments mentioned in Table 3, metrics are obtained from the models (Loss, mAP), which are generated by an eval_results script found in the repository. These metrics are stored as events in files with extension *.tfevents, in addition to other metrics calculated on the computer (confusion matrix, Precision–Recall). iii. Results: All metrics are visualized on a computer using a Python (version 3.9.4) virtual environment that has TensorBoard installed, whose tool enables the visualization and reading of all event files extracted from the Jetson.

**Figure 9:**
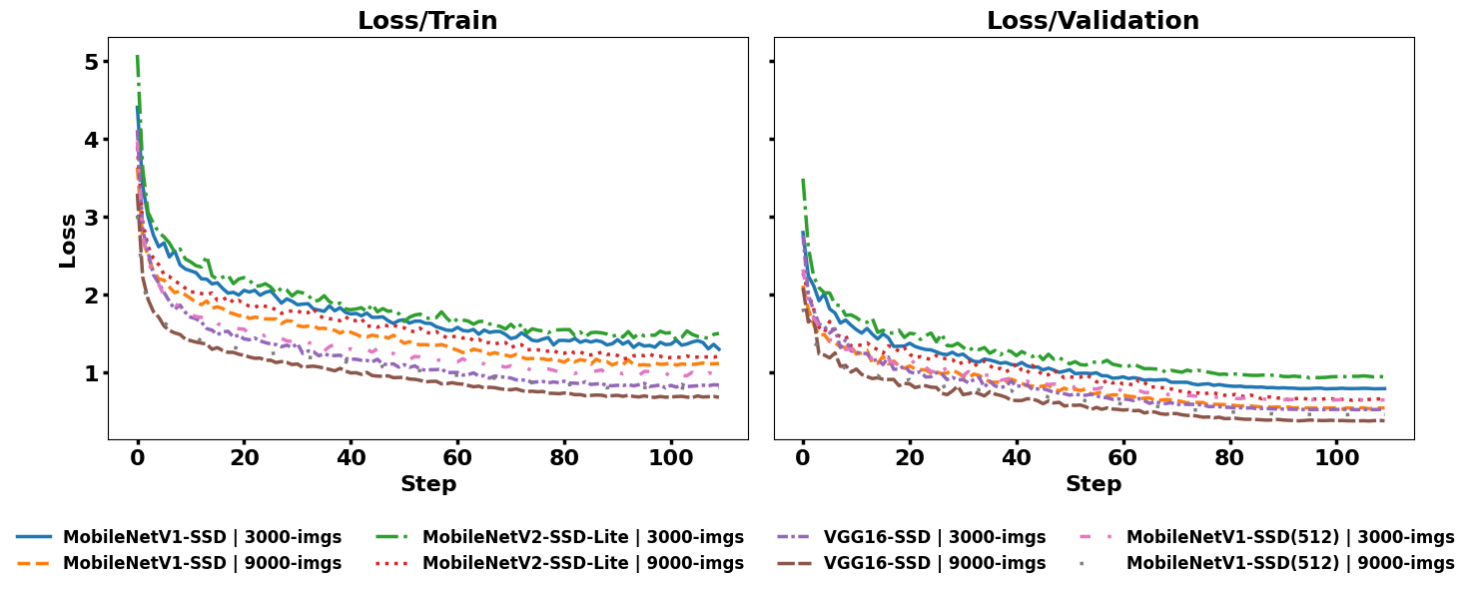
Loss/train and loss/validation.

In Figure 9, the loss curves for the train and validation datasets are shown, obtaining six curves in total for each case. A trend is observed in the curves that indicates that the models learn important patterns from the dataset. Considering that the VGG16-SSD model presents the lowest loss values in validation, this is related to its capacity to extract more features. On the other hand, MobileNetV2-SSD-Lite has higher and less stable loss during training. Furthermore, there are no signs of overfitting, as the validation curves do not diverge from the training curves.

### 4.1. Metrics

The work developed addresses an object detection problem, for which the most commonly used metric for this type of task is mean Average Precision (mAP). This requires several preliminary steps: i. obtain the probability of each object associated with a bounding box, which has been correctly identified in the data labeling stage; ii. calculate Precision and Recall; iii. calculate the PR curve for each class; iv. calculate the Average Precision (AP) for each class; v. calculate the average of AP across different classes.

**Figure 10:**
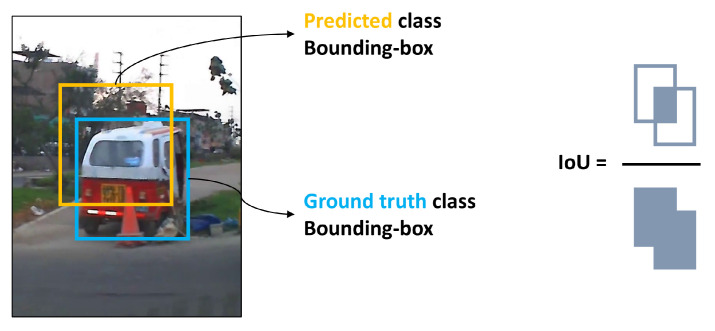
Intersection over Union (IoU) score.

The probability associated with each bounding box is calculated by dividing the area of overlap by the area of the union, as shown in the following equation:

(1) $$\mathrm{IoU}=\frac{B_{\mathrm{Ground}\;\mathrm{Truth}}\cap B_{\mathrm{Predicted}}}{B_{\mathrm{Ground}\;\mathrm{Truth}}\cup B_{\mathrm{Predicted}}}$$

where $B_{\mathrm{Ground}\;\mathrm{Truth}}$ is the position of the bounding box of a labeled class and $B_{\mathrm{Predicted}}$ is the predicted position of the bounding box. It is presented graphically in Figure 10.

This probability, IoU, is used to define the correct prediction (True Positive), which must be greater than a threshold. For object detection problems, a value of 0.5 is usually accepted; if the prediction value is greater, it is a True Positive (TP), but if it is lower, it is considered a False Positive (FP).

For each class, it is possible to obtain the Precision and Recall metrics using the values of TP and FP, in addition to False Negatives (FN), using the following equations:

(2) $$\mathrm{Recall}=\frac{\mathrm{TP}}{\mathrm{TP}+\mathrm{FN}};\;\mathrm{Precision}=\frac{\mathrm{TP}}{\mathrm{TP}+\mathrm{FP}}$$

#### 4.1.1. Confusion Matrix

**Figure 11:**
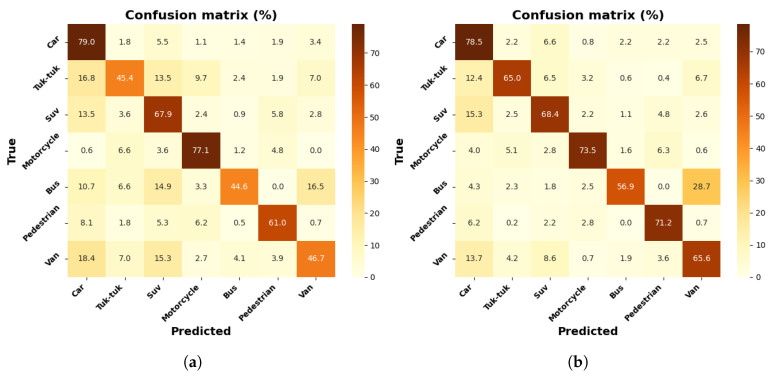
Results with MobileNetV1-SSD model: (**a**) confusion matrix with a 3000 frame dataset; (**b**) confusion matrix with a 9000 frame dataset.

Figure 11a,b show row-normalized confusion matrices for the MobileNetV1-SSD model in the object detection task with the collected dataset and the dataset with increment through contrast adjustment, with seven labeled classes. The results indicate an improvement with data increment, notably in the Tuk-tuk class, with an increase of up to 19.6%, according to the percentages reported in the matrices for all classes, with the exception of the Car and Motorcycle classes. The most significant class achieves 79% for the Car class, with the dataset of 3000 images.

**Figure 12:**
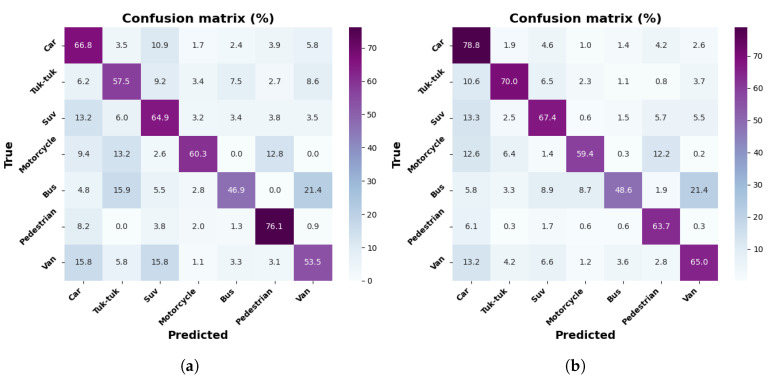
Results with MobileNetV2-SSD-Lite model: (**a**) confusion matrix with 3000 frames dataset; (**b**) confusion matrix with 9000 frames dataset.

Figure 12a,b show row-normalized confusion matrices for the MobileNetV2-SSD-Lite model, with the collected dataset and the dataset with increment through contrast adjustment. The results indicate an improvement with data increment, notably in the Tuk-tuk class, with an increase of up to 12.5%, according to the percentages reported in the matrices for all classes, with the exception of the Suv and Pedestrian classes. The most significant class achieves 78.8% for the Car class, with the dataset of 9000 images.

**Figure 13:**
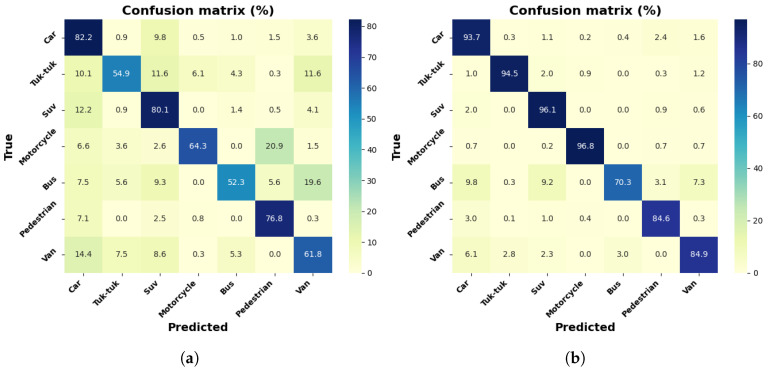
Results with VGG16-SSD model: (**a**) confusion matrix with 3000 frames dataset; (**b**) confusion matrix with 9000 frames dataset.

Figure 13a,b show row-normalized confusion matrices for the VGG16-SSD model, with the collected dataset and the dataset with increment through contrast adjustment. The results indicate an improvement with data increment, notably in the Tuk-tuk class, with an increase of up to 39.6%, according to the percentages reported in the matrices for all classes. The most significant class achieves 96.8% for the Motorcycle class, with the dataset of 9000 images.

**Figure 14:**
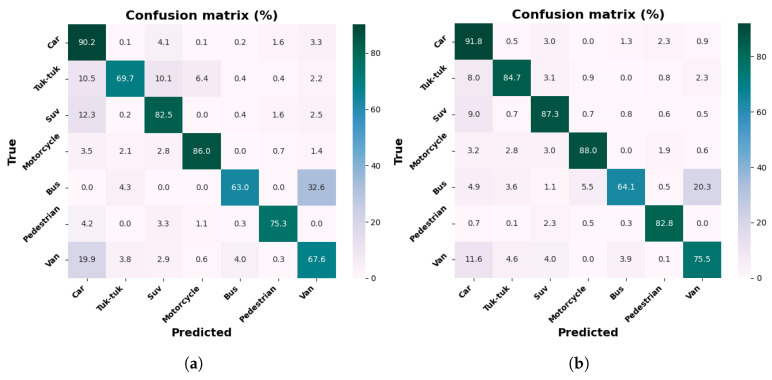
Results with MobileNetV1-SSD model (512 × 512): (**a**) confusion matrix with 3000 frames dataset; (**b**) confusion matrix with 9000 frames dataset.

Figure 14a,b show row-normalized confusion matrices for the MobileNetV1-SSD (512 × 512) model, with the collected dataset and the dataset with increment through contrast adjustment. The difference with the previous model is the image size in the model’s input layer, adjusted to a resolution of 512×512. The results indicate an improvement with data increment, notably in the Tuk-tuk class, with an increase of up to 15%, according to the percentages reported in the matrices for all classes. The most significant class achieves 91.8% for the Car class, with the dataset of 9000 images.

The confusion matrices show, in general, that the Car class presents high levels of accuracy, which is due to its shapes being more defined and clear than those of other elements. On the other hand, the Tuk-tuk and Motorcycle classes show higher confusion rates, especially in the MobileNetV1-SSD (300 × 300) and MobileNetV2-SSD-Lite models, due to their reduced size. The models trained with the augmented dataset show improvements, which evidences that contrast adjustment increases detection under lighting variations.

#### 4.1.2. Precision–Recall

The Precision–Recall (PR) curve is used to evaluate the performance of object detection models, whose presented graphs show the confidence for each class. A detection model is good if Precision remains high when Recall increases. The PR curve plots Precision on the Y-axis and Recall on the X-axis for different threshold values of the classifier.

**Figure 15:**
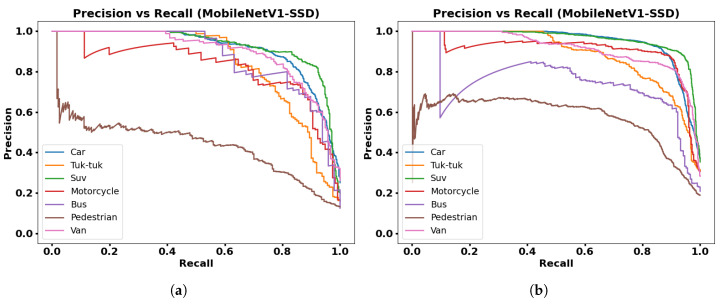
Resultswith MobileNetV1-SSD model: (**a**) Precision vs. Recall with 3000 frames dataset; (**b**) Precision vs. Recall with 9000 frames dataset.

**Figure 16:**
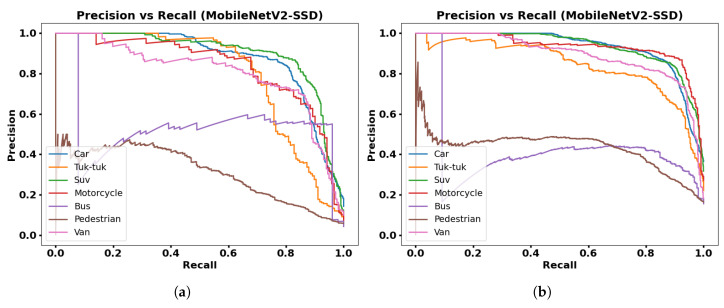
Results with MobileNetV2-SSD-Lite model: (**a**) Precision vs. Recall with 3000 frames dataset; (**b**) Precision vs. Recall with 9000 frames dataset.

**Figure 17:**
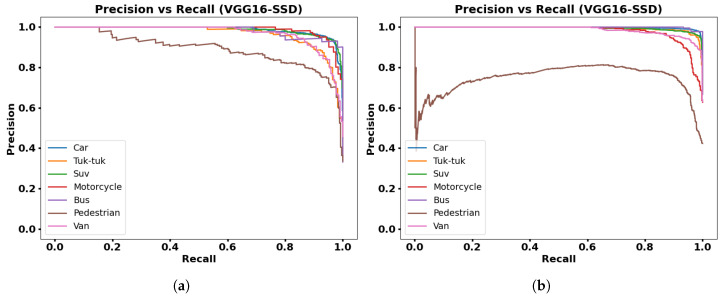
Results with VGG16-SSD model: (**a**) Precision vs. Recall with 3000 frames dataset; (**b**) Precision vs. Recall with 9000 frames dataset.

**Figure 18:**
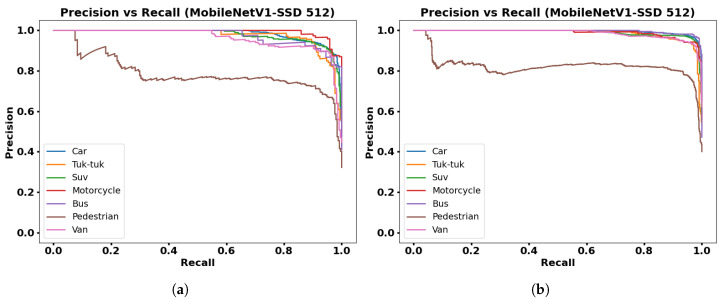
Results with MobileNetV1-SSD model (512 × 512): (**a**) Precision vs. Recall with 3000 frames dataset; (**b**) Precision vs. Recall with 9000 frames dataset.

Figure 15, Figure 16, Figure 17 and Figure 18 show PR graphs for the MobileNetV1-SSD, MobileNetV2-SSD-Lite, VGG16-SSD, and MobileNetV1-SSD (512 × 512) models, respectively. Additionally, subfigure (a) in all figures shows results using the collected dataset of 3000 images, while subfigure (b) shows results with the augmented dataset (9000 images) through contrast adjustment.

The PR curves show that the Car, Suv, and Motorcycle classes have curves with high precision, which indicates detections with low false positive rates. The VGG16-SSD model has more stable PR curves with larger area, which indicates its greater feature extraction capacity.

#### 4.1.3. Average Precision

To measure the quality of the predictions from the retrained models for each of the defined classes, the Average Precision (AP) metric is considered, which combines the percentage of correct detections and the percentage of real objects that were detected by the models used. This metric is calculated as the area under the PR curve, calculated previously, for a specific class.

(3) $$\mathrm{AP}=\int_{0}^{1}p(r)\;dr$$

where p(r) is Precision as a function of Recall.

**Figure 19:**
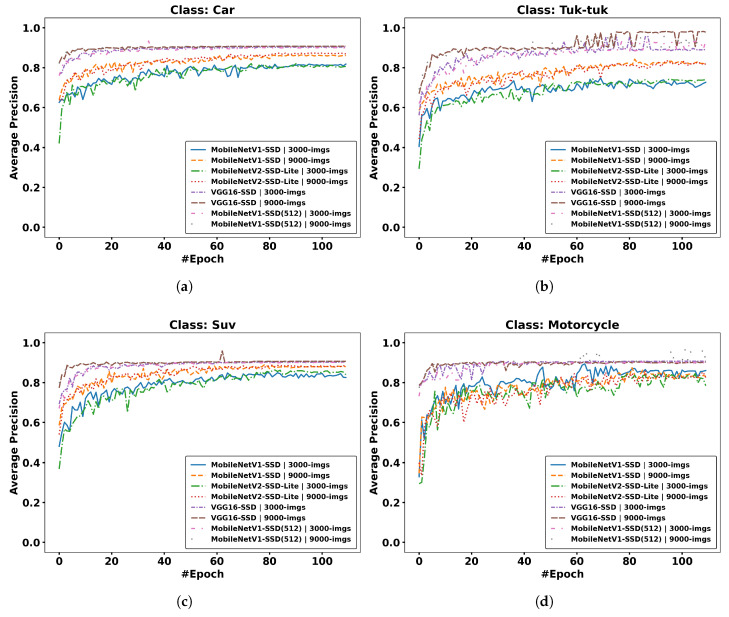

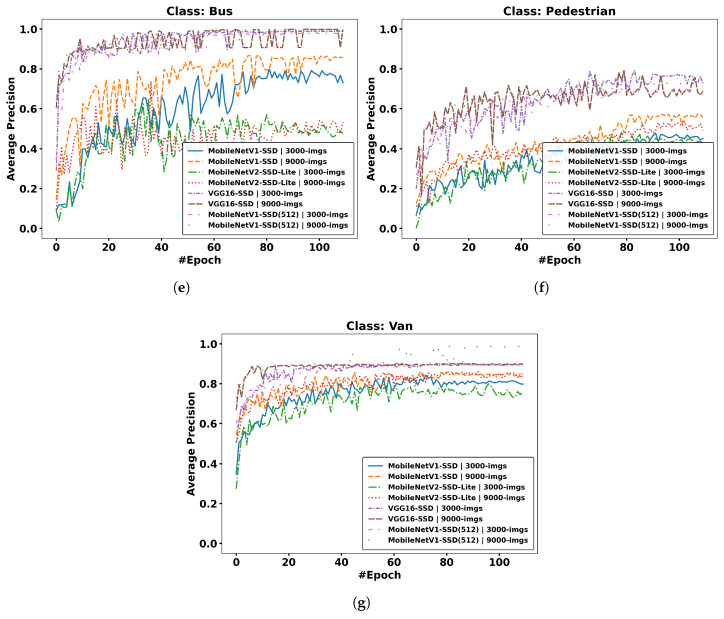
Results per class, Average Precision vs. epoch: (**a**) Car; (**b**) Tuk-tuk; (**c**) Suv; (**d**) Motorcycle; (**e**) Bus; (**f**) Pedestrian; (**g**) Van.

In Figure 19, the AP result is shown in each subfigure (seven classes) for each defined class; furthermore, in each of them are all the considered models and the two datasets used, resulting in a total of eight curves for each class.

In Figure 19, it is observed that subfigures (a), (c), and (d), belonging to the Car, Suv, and Motorcycle classes, respectively, present higher AP across all curves of all explored models. In subfigure (b), belonging to the Tuk-tuk class, a higher AP is seen for some of the models compared to other subfigures. Subfigure (e), of the Bus class, presents the most variable AP across the explored models compared to the other classes. On the other hand, subfigure (f), belonging to the Pedestrian class, presents a lower AP compared to the rest of the classes shown in the other subfigures. Finally, subfigure (g), of the Van class, presents a higher AP for a specific model.

The Car, Suv, and Motorcycle classes achieve high AP values quickly. Furthermore, the augmented dataset accelerates convergence in most classes, due to the greater data variability generated by contrast adjustment.

#### 4.1.4. Mean Average Precision

**Figure 20:**
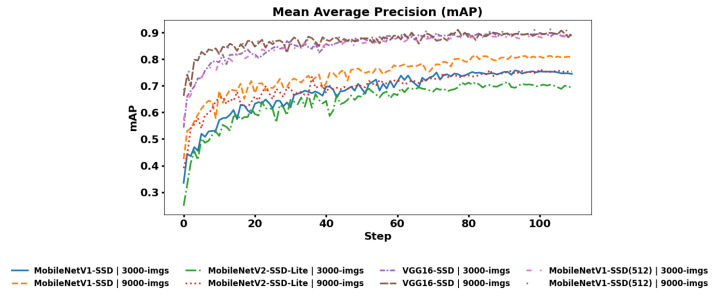
Mean Average Precision for all classes for each model.

This metric is the average of all the previously calculated AP (Average Precision) for each class defined in the dataset:

(4) $$\mathrm{mAP}=\frac{1}{N}\sum_{i=1}^{N}{\mathrm{AP}}_{\left(i\right)}$$

where *N* is the number of defined classes. In Figure 20, the mAP metric of all the initially defined classes is shown, where each curve in the graph is one of the fine-tuned models used with the collected dataset and the dataset increased by contrast adjustment.

According to the mAP metric reported in the graph, it is shown that the VGG16-SSD model is superior to the MobileNetV1-SSD and MobileNetV2-SSD-Lite models because it uses more parameters and convolutional layers, which enables information capable of discriminating elements with variable shapes (such as the Tuk-tuk class) to be obtained, generating more detailed feature maps. This generates an increase in computational cost that increases the training time for the NVIDIA Jetson device (as reported in Table 4). However, the MobileNetV1-SSD (512 × 512) model with an input layer of 512 × 512 presents results similar to the performance of the VGG16-SSD model.

In the case of MobileNetV2-SSD-Lite, it generates irregular behavior when the data to be evaluated have high shape variability and does not capture edges or shapes in small vehicles. On the other hand, MobileNetV1-SSD uses more stable convolutional stages that allow more spatial information, complex environments, and variable lighting conditions to be identified, which is why this model presents better performance. However, it also presents a higher use of computational resources.

### 4.2. Test on Platform

All models have been fine-tuned on the platform, as shown in Table 4, considering the lowest loss value; the validation set for both datasets of all the models used, showing the mAP value; the epoch number in which this value was found; and the time it took the platform to train up to that epoch.

Note that the superior performance of VGG16-SSD with 90.74% is again confirmed, but it requires a longer training time than the others of 1.64 days, however the MobileNetV1-SSD (512 × 512) model presents a considerably very close mAP value (90.44%); even with the unaugmented dataset (88.47%) and with a much shorter training time (8.02 h), it is possible for it to achieve very similar metrics of 88.79% compared to the VGG16-SSD model.

**Figure 21:**
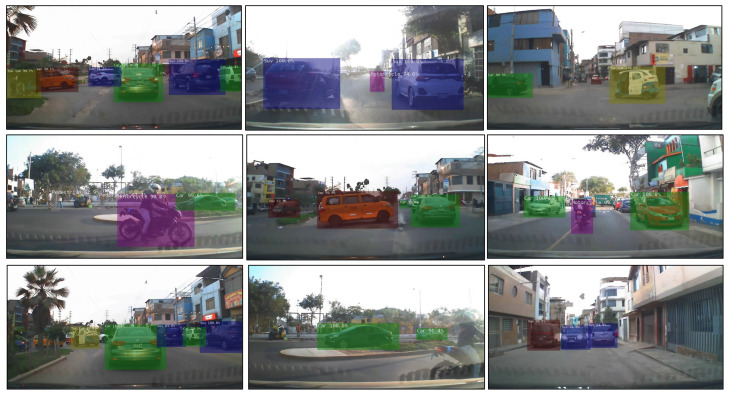
Object detection using MobileNetV1-SSD (512 × 512) on an embedded platform.

The trained MobileNetV1-SSD (512 × 512) model is implemented on the Jetson Orin NX 16 GB using the available documentation and libraries from NVIDIA. For the detection test of this trained model, DetecNet is used, loading the previously exported model in *.onnx format, and indicating the input data or source (images, videos or camera) and the output path to save the images once the detections have been performed. Some examples of object detection of the different classes that worked with the aforementioned model are shown in Figure 21.

### 4.3. Energy Consumption on the Jetson Platform

Energy consumption readings were obtained using the software tool available from NVIDIA, based on data obtained by the INA3221 current sensor, which is embedded in the Jetson Orin NX 16 GB. Measurements were performed during the detection stage of the model loaded and implemented with DetecNet, using the validation image dataset as the input. During a determined time period, while performing inference for each model, all readings were recorded in a *.csv file, which was extracted from the embedded platform. The consumption of VDD_IN (total power consumption); VDD_CPU_GPU_CV (combined CPU, GPU, and CV power rail); and VDD_SOC (SOC power rail) was monitored. All of these readings were averaged to obtain the data displayed in Table 5, including tests with both datasets.

Note that the total power consumption readings VDD_IN (≈7.3 W, ≈7.4 W, and ≈7.5 W) for each explored model (MobileNetV2-SSD-Lite, MobileNetV1-SSD-300 × 300, and MobileNetV1-SSD-512 × 512) increase in order, as does the mAP metric (75%, 80%, and 90%) achieved by each of the implemented models.

### 4.4. Comparative Performance with YOLO-v8-Nano

TensorRT, a C++ library, is used to facilitate inference on NVIDIA GPU units. TensorRT takes a trained model and generates an optimized engine that performs inference for that network [41]. A comparison of latency and size is made between the best model obtained (MobileNetV1-SSD) and the YOLO (You Only Look Once) model in version 8 Nano, presented in Ref. [42], using an FP32 engine for both models on the Jetson Orin NX. Additionally, the number of parameters of each model and the mAP metric for the dataset of 9000 frames with an input layer size of 512 × 512 for both models are shown in Table 6.

YOLO-v8-Nano shows good results in terms of accuracy, in addition to being lightweight in size and also showing lower latency when performing inference. However, despite having 60% fewer parameters compared to MobileNetV1-SSD, the difference in latency is very close. All latency values, as well as the FPS number shown (calculated as 1000/Latency(median)), correspond only to the pure inference of each of the object detection models.

In implementation within an embedded system, like the present work, in addition to considering the inference time of the model, it is necessary to consider additional times such as pre and post-processing. A comparison of both models is shown in Table 7. For this activity, in the case of MobileNetV1-SSD, DetecNet is used to load the model, which creates an FP16 engine; in the case of YOLO-v8-Nano, TensorRT has been used to generate an engine of the same type (FP16). DetecNet shows the visualizations of the images with the generated bounding-boxes immediately after performing the inference, while YOLO does not. Therefore, for a fairer comparison, the visualization stage (0.14 ms) has been omitted, and three stages have been considered: pre-process, network, and post-process.

The main reason for the difference in time between both models is that the YOLO source code is written in Python, while DetecNet uses C++ and CUDA, which means it takes less time in the pre and post-processing stages.

Finally, a comparison of the energy consumption is shown in Table 8, and as in the previous table, the visualization stage has also been omitted.

MobileNetV1-SSD, in the FP16 engine, presents a consumption of approximately 7.2 W, being lower than the value reported in Table 5, due to the absence of the visualization stage. On the other hand, YOLO presents a consumption of approximately 7.6 W, showing that the SSD-based model has slightly more energy-efficient consumption compared to YOLO.

An energy report and analysis regarding YOLO-v8 applied in different engines (FP32, FP16, and INT8) and on the same platform used in this study (NVIDIA Jetson Orin NX) can be found in [34].

### 4.5. Comparative Performance with the Literature

Finally, a comparison with similar works with computer vision models, for object detection, implemented on an NVIDIA embedded platform is performed, which is presented in Table 9.

The results demonstrate that the MobileNetV1-SSD (512 × 512) model implemented in this work achieves a significantly higher mAP compared to the other models on the NVIDIA Jetson platform.

## 5. Conclusions

In this work, the study and implementation of vehicle and person detection models based on deep learning networks, such as the SSD network, is proposed, with the aim of contributing to the creation of autonomous driver assistance systems, in a real-world environment on the streets of Lima, Peru. The computer vision models have been implemented on an embedded platform with GPU, specifically the NVIDIA Jetson Orin NX card. In this study, a custom dataset has been collected, and data augmentation has been performed with contrast adjustment on the constructed dataset. The training of the models has been carried out on the same platform, considering the tools available from NVIDIA and some models available within the documentation of previous works. The times taken for the fine-tuning of each model have been reported, as well as the reliability metrics of the system, using the collected dataset and the augmented dataset. In future work, other data augmentation techniques and the capture of a larger dataset, considering possible changes in weather conditions, will be explored. Additionally, studies on the power consumption monitoring of the execution of each implemented models will be carried out.

This work contributes to the exploration and implementation of intelligent models in embedded applications with high computational performance and low power consumption, specifically in real-time tasks, where detection accuracy and speed are paramount, as in the case of driver assistance systems.

## Figures and Tables

**Table 1:** Distribution of classes in the dataset.

#	Class	No. of Objects
1	Car	4924
2	Tuk-tuk	1101
3	SUV	3082
4	Motorcycle	822
5	Bus	430
6	Pedestrian	2201
7	Van	1452
	Total	14,012

**Table 2:** Class distribution.

#	Class		Dataset (3000)			Dataset (9000)	
		Train	Test	Val.	Train	Test	Val.
1	Car	3297	772	855	10,064	2268	2440
2	Tuk-tuk	745	174	182	2188	531	584
3	SUV	2072	443	573	6165	1449	1650
4	Motorcycle	555	126	141	1679	390	397
5	Bus	272	55	103	857	225	208
6	Pedestrian	1500	312	389	4498	1057	1048
7	Van	966	226	260	2988	629	739

**Table 3:** Arguments to train models.

#	Argument	Value(s)
1	–dataset-type	voc
2	–net	mb1-ssd; mb2-ssd-lite; vgg16-ssd
3	–resolution	300; 512
4	–pretrained-ssd	mobilenet-v1-ssd-mp-0_675.pth; mb2-ssd-lite-mp-0_686.pth; vgg16-ssd-mp-0_7726.pth
5	–batch-size	4
6	–epochs	110
7	–validation-epochs	1
8	–debug-steps	10
9	–use-cuda	True

**Table 4:** Report on the NVIDIA Jetson platform.

Dataset	Model	# Step (Epoch)	Time	Loss/Val	mAP
	MobileNetV1-SSD (300 × 300)	98	7.212 h	0.7871	75.43
3000 frames	MobileNetV1-SSD (512 × 512)	99	8.019 h	0.6413	88.47
	MobileNetV2-SSD-Lite	95	7.966 h	0.9252	70.71
	VGG16-SSD	106	13.19 h	0.5194	88.79
	MobileNetV1-SSD (300 × 300)	101	1.105 days	0.5272	80.44
9000 frames	MobileNetV1-SSD (512 × 512)	106	1.103 days	0.4539	90.44
	MobileNetV2-SSD-Lite	103	1.029 days	0.6388	75.17
	VGG16-SSD	106	1.644 days	0.3734	90.74

**Table 5:** Report of the energy consumption.

Model	VDD Type	Voltage	Current	Power
MobileNetV1-SSD (300 × 300)	IN	5087.80 mV	1449.86 mA	7374.64 mW
	CPU_GPU_CV	5072.86 mV	442.60 mA	2244.70 mW
	SOC	5080.00 mV	320.54 mA	1627.79 mW
MobileNetV1-SSD (512 × 512)	IN	5088.00 mV	1481.60 mA	7538.90 mW
	CPU_GPU_CV	5072.00 mV	461.60 mA	2340.68 mW
	SOC	5080.00 mV	324.13 mA	1646.11 mW
	IN	5088.00 mV	1428.31 mA	7263.58 mW
MobileNetV2-SSD-Lite	CPU_GPU_CV	5074.77 mV	431.38 mA	2190.00 mW
	SOC	5080.92 mV	318.15 mA	1615.77 mW

**Table 6:** Performance of inference comparison: MobileNetV1-SSD vs. YOLO-v8-Nano.

Model	#Parameters	Size (Engine)	Latency (Mean)	Latency (Median)	Latency (P-99%)	FPS	Metric (Value)
MobileNetV1-SSD	7.48 M	30.6 MB	≈12.446 ms	≈12.380 ms	≈13.987 ms	≈81	mAP = 90.4%
YOLO-v8n	3.03 M	13.3 MB	≈11.134 ms	≈10.927 ms	≈15.740 ms	≈92	mAP = 91.7%

**Table 7:** Timing report comparison: MobileNetV1-SSD vs. YOLO-v8-Nano.

Stages	MobileNetV1-SSD	YOLO-v8-Nano
Pre-Process	≈0.1 ms	≈5.4 ms
Network	≈9.0 ms	≈7.8 ms
Post-Process	≈0.2 ms	≈14.9 ms
Total Time	≈9.3 ms	≈28.1 ms

**Table 8:** Report of the energy consumption: MobileNetV1-SSD vs. YOLO-v8-Nano.

		MobileNetV1-SSD			YOLO-v8-Nano	
VDD Type	Voltage	Current	Power	Voltage	Current	Power
IN	5088.00 mV	1417.71 mA	7212.86 mW	5088.00 mV	1468.00 mA	7560.38 mW
CPU_GPU_CV	5073.71 mV	424.57 mA	2153.71 mW	5073.00 mV	437.00 mA	2221.50 mW
SOC	5080.00 mV	316.00 mA	1607.43 mW	5080.00 mV	333.00 mA	1691.13 mW

**Table 9:** Performance comparison with the literature.

Reference	Model	Embedded-Plataform	Classes	Metric (Value)
Barba-Guaman et al., 2020 [43]	PedNet	Jetson Nano	car, pedestrian	Acc. = 78.71%
Farooq et al., 2021 [44]	YOLO-v5	Jetson Nano	bike, bus, bycicle, car, dog, person, pole	mAP = 85.5%
Elmanaa et al., 2023 [45]	YOLO-v7-tiny	Jetson Nano	car, bus, motorcycle, truck	mAP = 80.1%
Liang et al., 2024 [46]	YOLO-v7-tiny (prune)	Jetson Xavier AGX	car, cyclist, pedestrian, tram, tricycle, truck	mAP = 72.5%
This work	MobileNetV1-SSD (512 × 512)	Jetson Orin NX	bus, car, motorcycle, pedestrian, suv, tuk-tuk, van	mAP = 90.44%

## Data Availability

The data presented in this study are openly available in Google Drive at https://drive.google.com/drive/folders/1phXCg5s1JgLBtfeS3lXWXR2nuC7QzqSl (accessed on 17 July 2025).
